# Supplementary material for: Transient auditory nerve demyelination as a new mechanism for hidden hearing loss
Source: Nat Commun. 2017 Feb 17;8:14487. doi: 10.1038/ncomms14487 (PMC5321746; doi:10.1038/ncomms14487)
Supplement: Supplementary Information — Supplementary Figures. [file ncomms14487-s1.pdf]

Supplementary Information

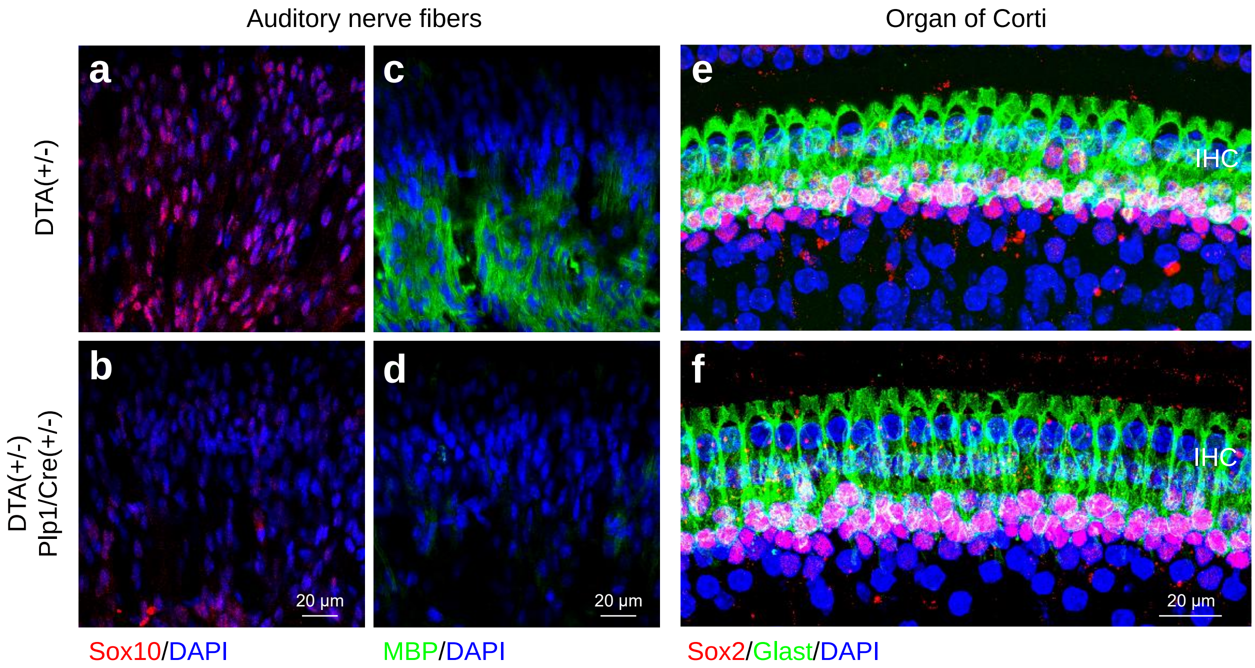

Supplementary Figure 1: Conditional DTA expression in *Plp1*-expressing cells of the mature cochlea leads to ablation of Schwann cells but not supporting cells.

DTA(+/-):Plp1/Cre(+/-) and control mice were injected intraperitoneally with tamoxifen P21-23 and cochlear tissues were analyzed at P30. (**a,b**) Tamoxifen injection induces loss of expression of the Schwann cell marker Sox10 along the auditory nerve fibers in *DTA(+/-):Plp1/Cre(+/-)* (**b**) but not in control mice (**a**). (**c,d**) Tamoxifen injection induces loss of of MBP expression along auditory nerve fibers in *DTA(+/-):Plp1/Cre(+/-)*(**d**) but not in control mice (**c**). (**e,f**) Tamoxifen injection P21-23 does not induce supporting cell loss in *DTA(+/-):Plp1/Cre(+/-)* mice. Sox2 and Glast were used as markers for the supporting cells (IPhC/IBCs) surrounding the IHCs.

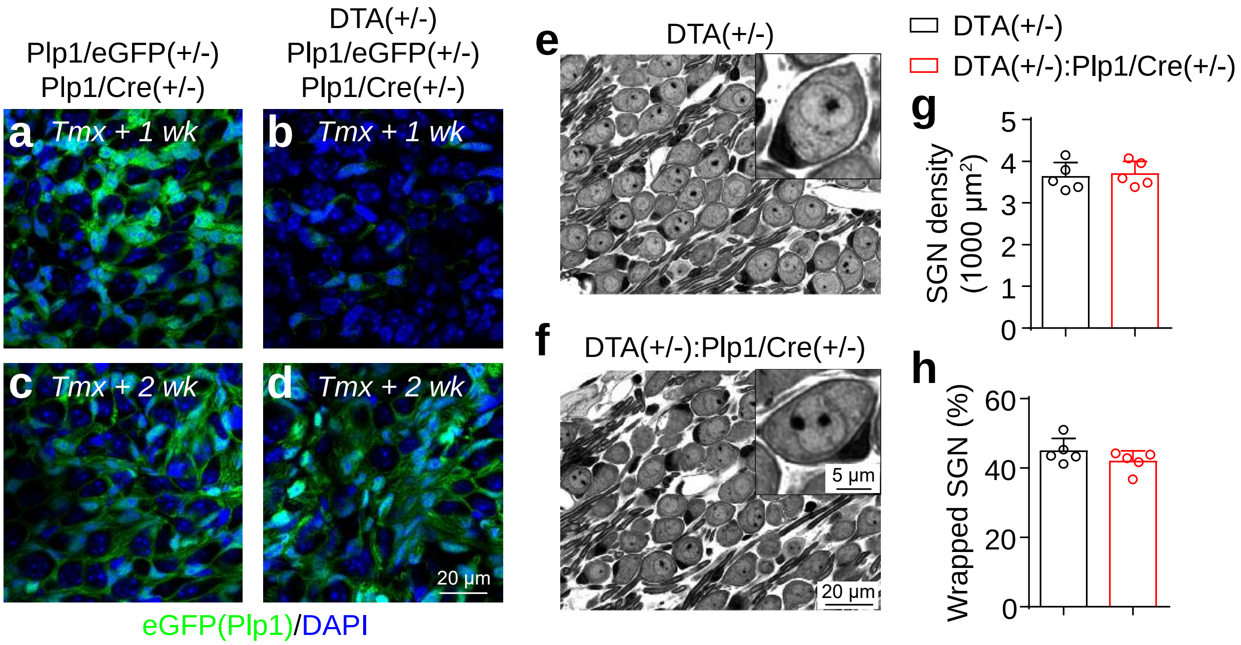

Supplementary Figure 2: Satellite cells are ablated and completely regenerated in *DTA(+/-):Plp1/Cre(+/-)* mice. (**a,b**) *Plp1*-expressing satellite cells marked by *Plp1/eGFP* are lost within 1 week after tamoxifen induction in *DTA(+/-):Plp1/eGFP(+/-):Plp1/Cre(+/-)* cochlea. (**c,d**) Satellite cells repopulate 2 weeks after tamoxifen induction. (**e-h**) SGN density and wrapping by satellite cells are normal at 16 weeks after DTA expression. (**e,f**) Light microscopic images of plastic sections through the spiral ganglio shows SGN cell bodies and their associated satellite cells in control (**e**) and *DTA(+/-):Plp1/Cre(+/-)* (**f**) cochlea. The inserts show high magnification views of single SGN cell body wrapped by optically-dense satellite cell. (**g,h**) Quantitative analysis shows that the density of SGN cell bodies (**g**) and the percentage of SGN cell bodies wrapped by satellite cells (**h**) are similar between *DTA(+/-):Plp1/Cre(+/-)* and control cochlea. *n* = 6 of each animal group.

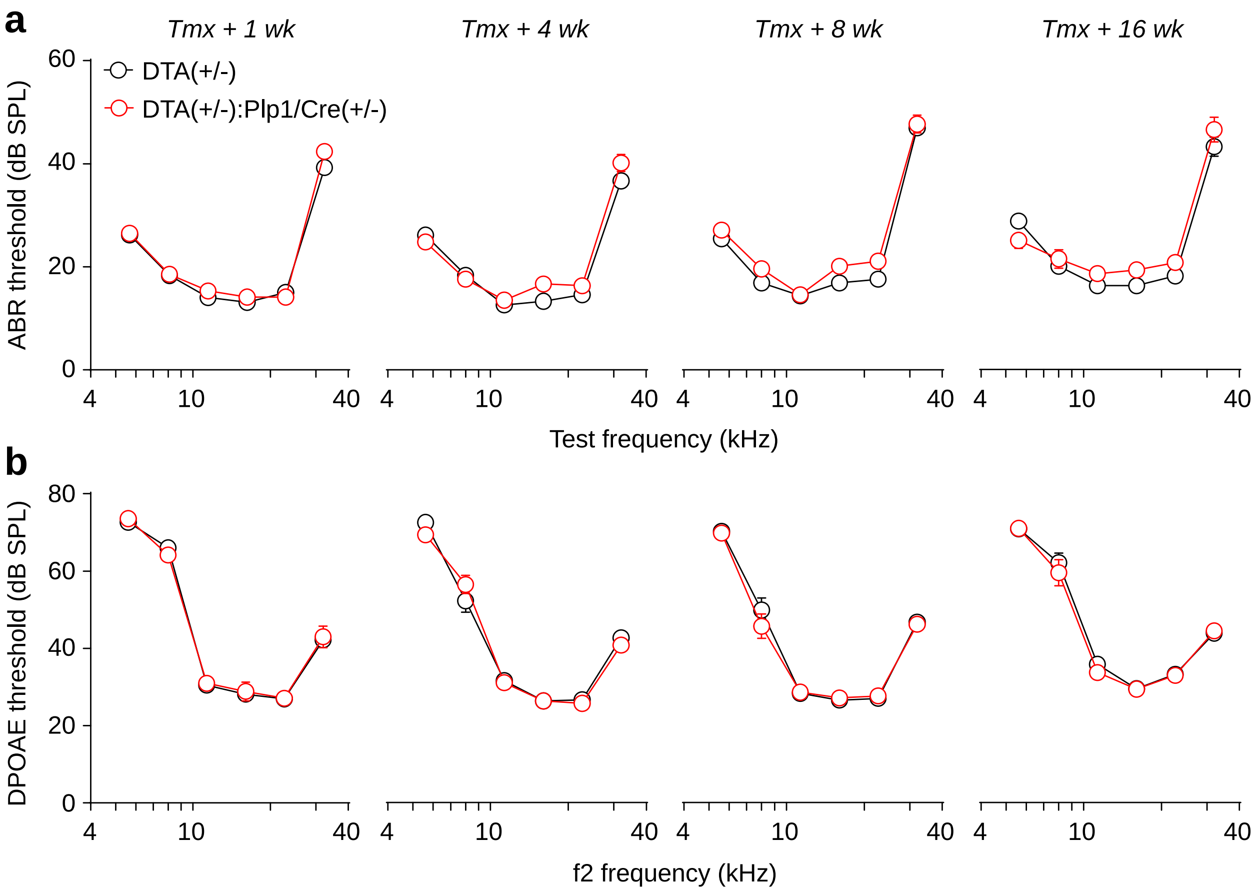

Supplementary Figure 3: Transient Schwann cell ablation does not affect auditory thresholds. Mice were injected with tamoxifen from P21-23 and ABR and DPOAE tests were performed 1, 4, 8 and 16 weeks later. ABR thresholds (**a**) and DPOAE thresholds (**b**) are unaffected at all timepoints in *DTA(+/-):Plp1/Cre(+/-)* mice at all frequencies. Tmx + 1 wk, *n* = 17-21; Tmx + 4 wk, *n* = 16-20; Tmx + 8 wk, *n* = 10-14; Tmx + 16 wk, *n* = 7-8 of each animal group.

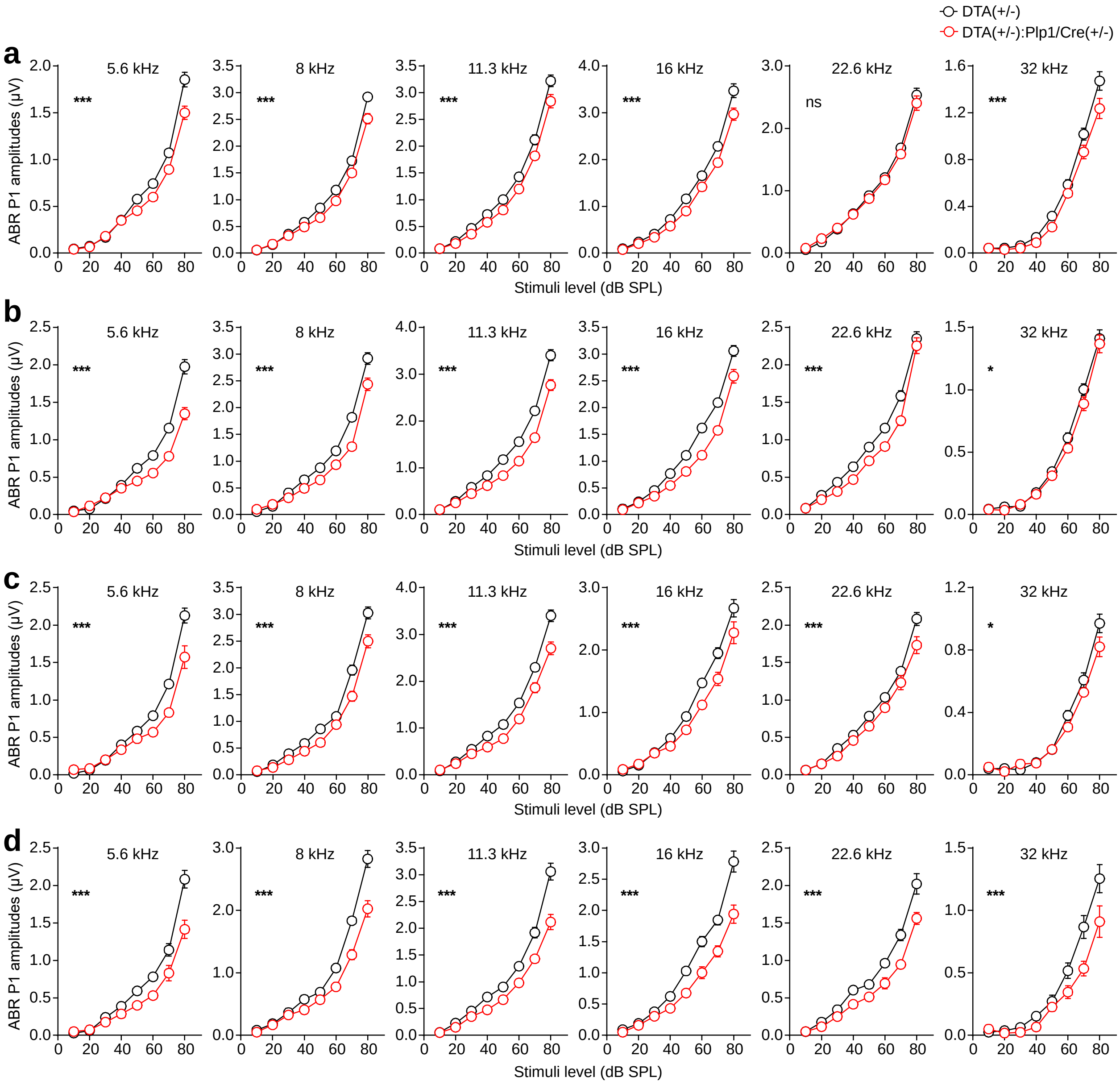

Supplementary Figure 4: Transient Schwann cell ablation impairs suprathreshold ABR peak 1 amplitude. Mice were injected with tamoxifen from P21-23 and ABR tests were performed (**a**) 1 week, (**b**) 4 weeks, (**c**) 8 weeks and (**d**) 16 weeks later. ABR peak 1 amplitude (μV) was plotted against the stimuli level at 5.6, 8, 11.3, 16, 22.6 and 32 kHz. Tmx + 1 wk, n = 17-21; Tmx + 4 wk, n = 16-20; Tmx + 8 wk, n = 10-14; Tmx + 16 wk, n = 7-8 of each animal group. * $p < 0.05$; *** $p < 0.001$ by two-way ANOVA.

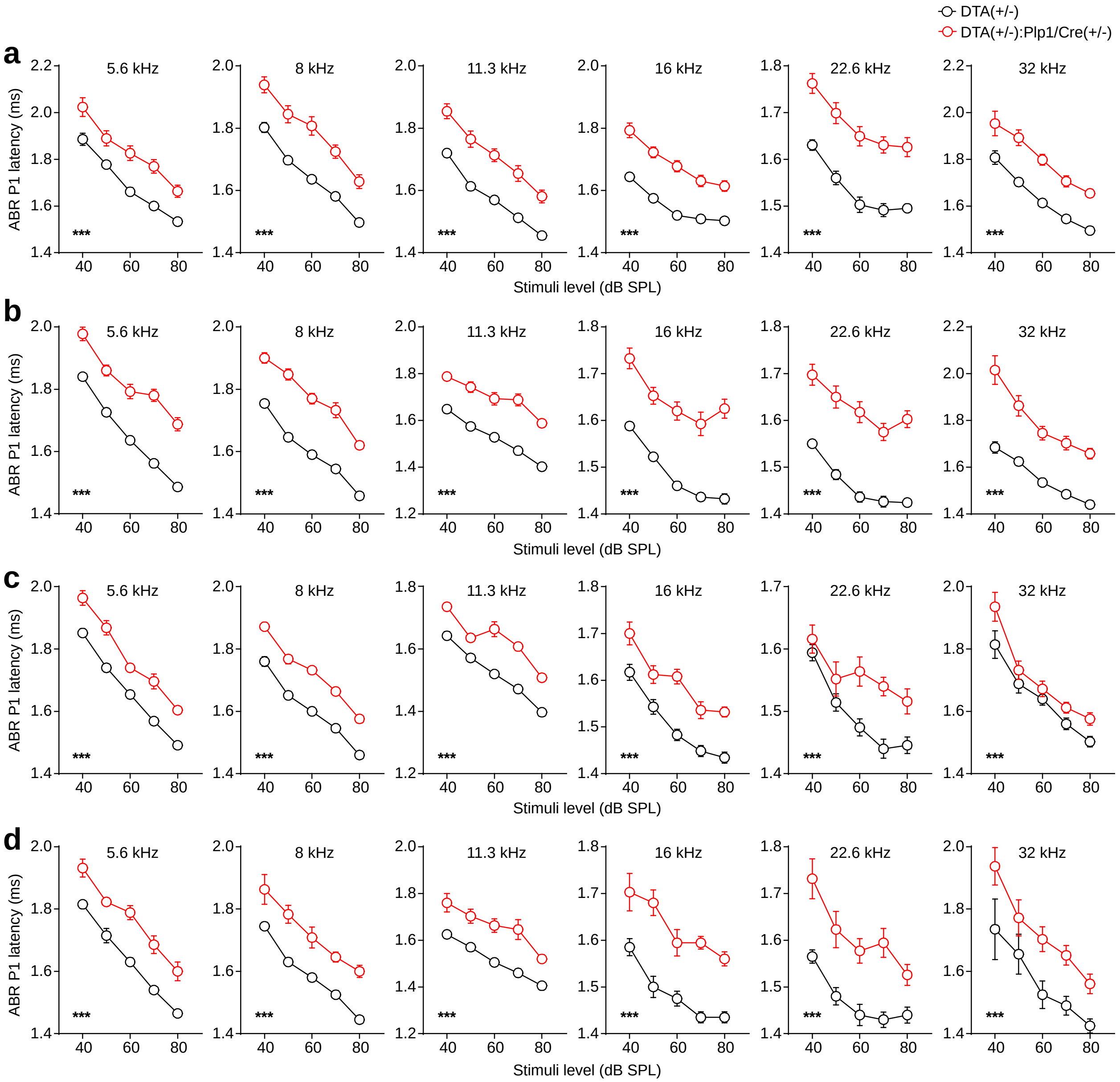

Supplementary Figure 5: Transient Schwann cell ablation impairs suprathreshold ABR peak 1 latency. Mice were injected with tamoxifen from P21-23 and ABR tests were performed (**a**) 1 week, (**b**) 4 weeks, (**c**) 8 weeks and (**d**) 16 weeks later. ABR peak 1 latency (ms) was plotted against the stimuli level at 5.6, 8, 11.3, 16, 22.6 and 32 kHz. Tmx + 1 wk, *n* = 17-21; Tmx + 4 wk, *n* = 16-20; Tmx + 8 wk, *n* = 10-14; Tmx + 16 wk, *n* = 7-8 of each animal group. *** $p < 0.001$ by two-way ANOVA.

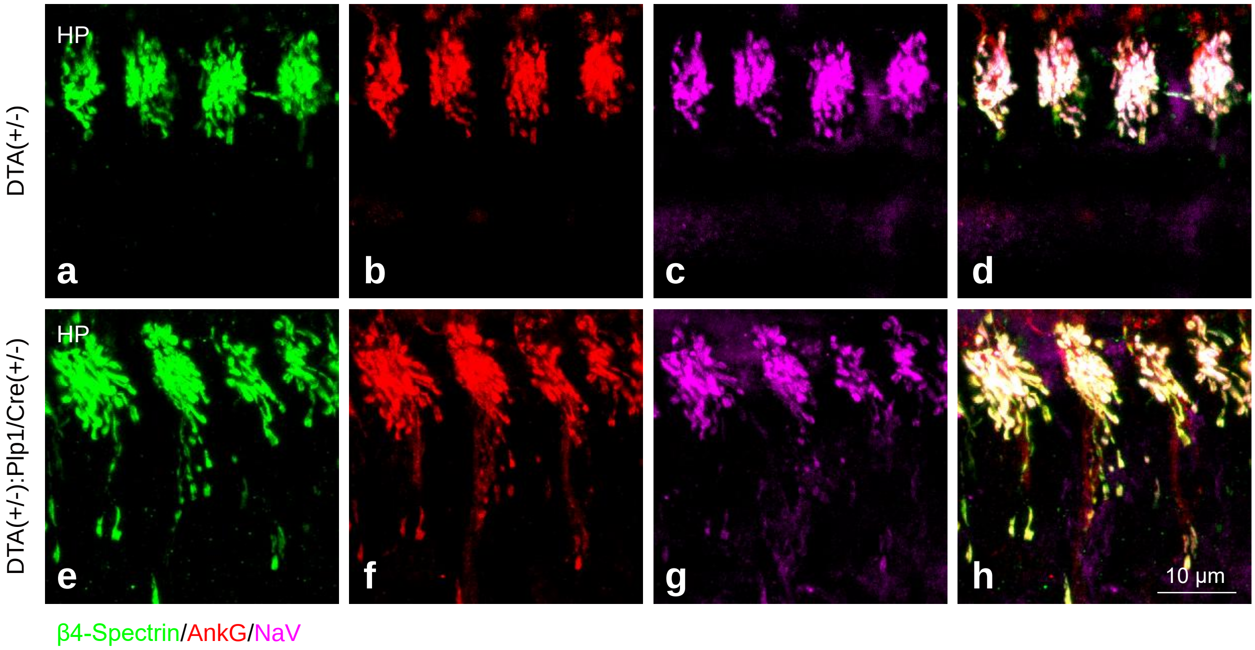

Supplementary Figure 6: Disruption of heminode organization after transient Schwann cell ablation. (**a-h**) Immunostaining for β4-spectrin (**a,e**), ankyrin G (**b,f**) and sodium channels (**c,g**) shows disorganization of heminodes in *DTA(+/-):Plp1/Cre(+/-)* cochlea (**e-h**) but not in control cochlea (**a-d**) 16 weeks after tamoxifen induction.

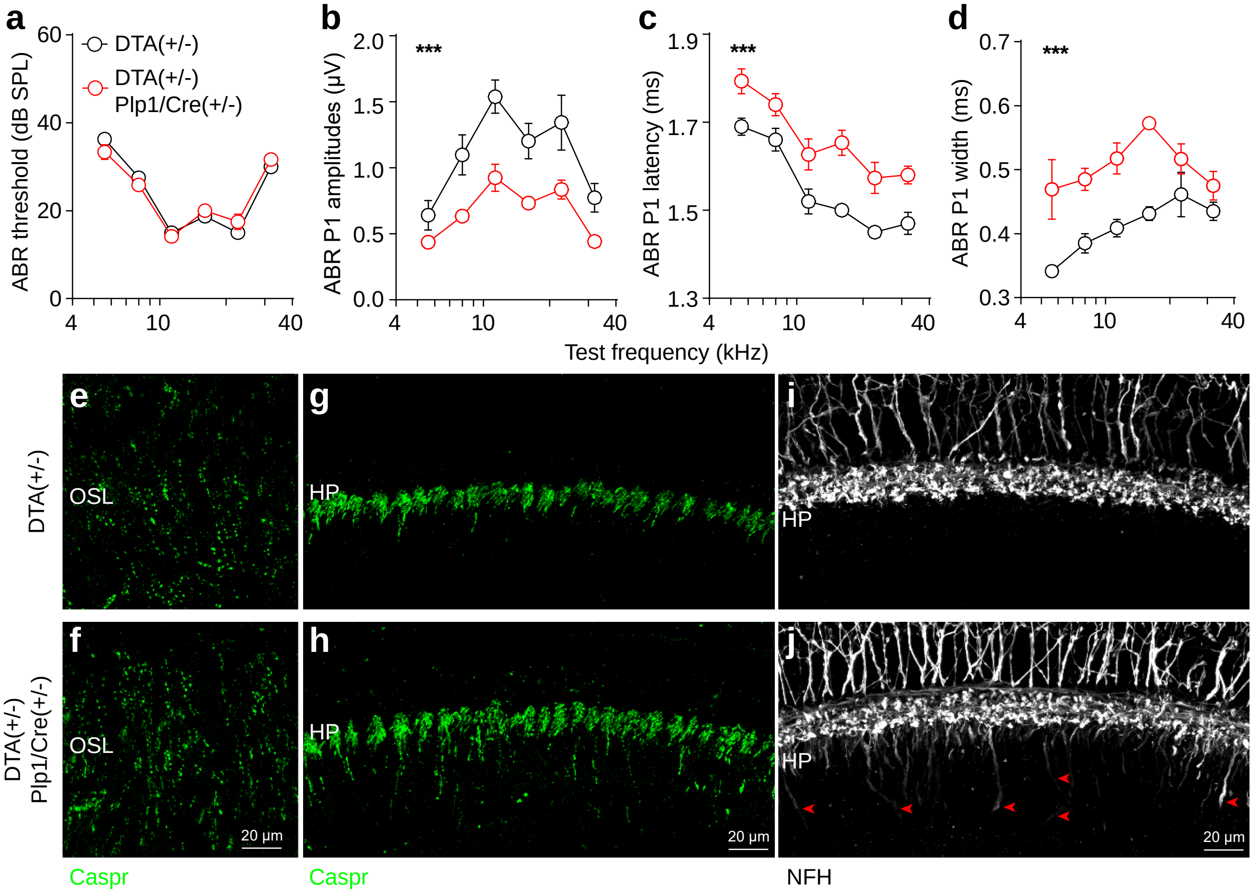

Supplementary Figure 7: Permanent auditory impairment and heminode disruption after transient Schwann cell ablation. *DTA(+/-):Plp1/Cre(+/-)* mice present with hidden hearing loss one year after tamoxifen induction as illustrated by the presence of normal ABR thresholds **(a)** but reduced ABR P1 amplitudes (**b**), increased ABR P1 latency (**c**) and increased ABR P1 width (**d**); *n* = 4-6 of each group. *** $p < 0.001$ by two-way ANOVA. Immunostaining show that nodes of Ranvier appear normal in the osseous spiral lamina (OSL) in *DTA(+/-):Plp1/Cre(+/-)* cochlea (**e, f**) 1 year after tamoxifen induction. In contrast, heminode disruption (**h**) and dysmyelinated nerve fibers remain present by the habenula perforata (**j**, arrow heads). **(g, i)** show images from control mice for comparison.

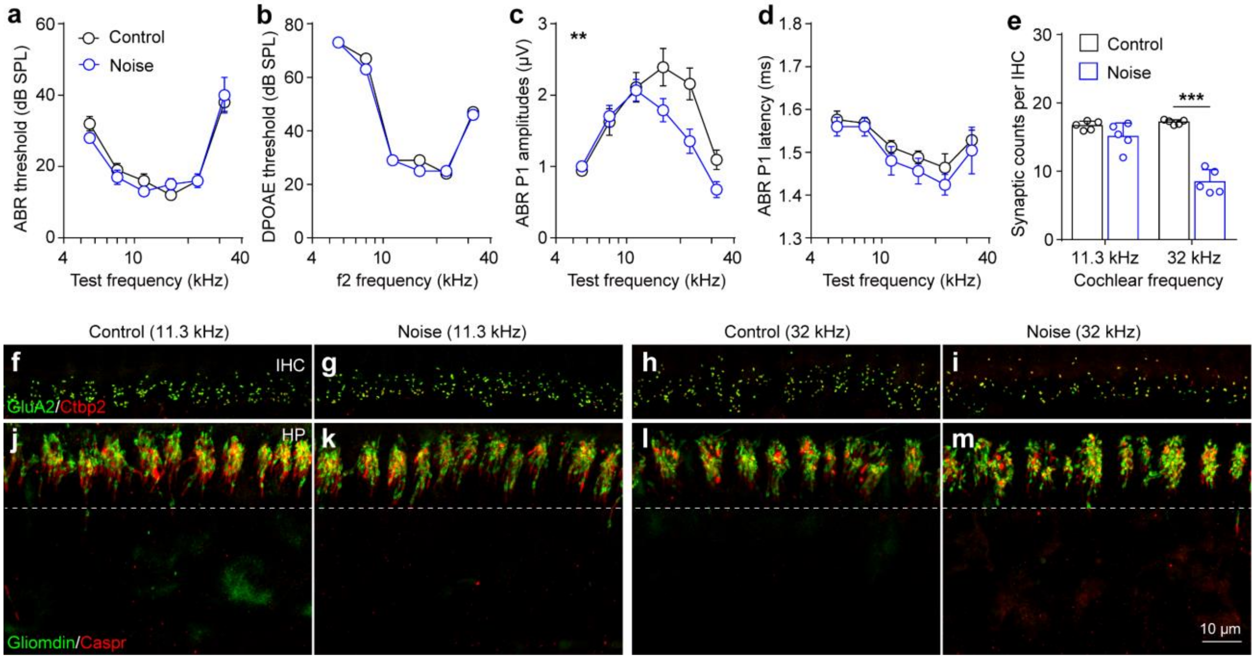

Supplementary Figure 8: Noise exposure that caused synaptopathic hidden hearing loss does not disrupt heminodes. (**a-d**) Mice exposed to 8-16 kHz noise at 100 dB SPL for 2 h display hidden hearing loss 2 weeks later as illustrated by the reduced ABR P1 amplitudes (**c**) without changes in ABR thresholds (**a**), DPOAE thresholds (**b**) or ABR P1 latencies (**d**). Sixteen week old wildtype FVB/N mice were used; *n* = 5 of each group. ** $p < 0.01$ by two-way ANOVA. (**e**) Noise exposure results in inner hair cell synapse loss at 32 kHz but not 11.3 kHz cochlear regions. **f-l** Representative images of ribbon synapse immunostaining of groups in **e**. *n* = 5 of each animal group. *** $p < 0.001$ by two-way ANOVA followed by Bonferroni's post-tests. (**j-m**) Representative images of nodal markers showing normal heminodes in noise-exposed mice.
